# The Prevalence of Asthma and Respiratory Symptoms among Cross-Country Skiers in Early Adolescence

**DOI:** 10.1155/2019/1514353

**Published:** 2019-09-15

**Authors:** E. Lennelöv, T. Irewall, E. Naumburg, A. Lindberg, N. Stenfors

**Affiliations:** ^1^Department of Public Health and Clinical Medicine, Division of Medicine, Umeå University, Östersund, Sweden; ^2^Department of Clinical Science, Paediatrics, Umeå University, Umeå, Sweden; ^3^Department of Public Health and Clinical Medicine, Division of Medicine, Umeå University, Sunderbyn, Sweden

## Abstract

**Objective:**

To determine the prevalence of asthma and respiratory symptoms among Swedish cross-country skiers in early adolescence in comparison to a population-based reference group of similar ages.

**Methods:**

A postal questionnaire on asthma, asthma medication, allergy, respiratory symptoms, and physical activity was distributed to Swedish competitive cross-country skiers aged 12–15 years (*n* = 331) and a population-based reference group (*n* = 1000). The level of asthma control was measured by the Asthma Control Test.

**Results:**

The response rate was 27% (*n* = 87) among skiers and 29% (*n* = 292) in the reference group. The prevalence of self-reported asthma (physician-diagnosed asthma and use of asthma medication in the last 12 months) and the prevalence of reported wheezing during the last 12 months were 23% and 25%, respectively, among skiers, which were significantly higher than the values reported in the reference group (12% and 14%). Skiers exercised more hours/week than the reference group. Among adolescents with self-reported asthma, neither the usage of asthma medications nor the level of asthma control according to the Asthma Control Test differed between skiers and the reference group.

**Conclusions:**

Adolescent competitive cross-country skiers have an increased prevalence of respiratory symptoms and asthma compared to nonskiers.

## 1. Introduction

Asthma is an inflammatory airway disease with episodes of narrowing airways leading to wheezing and shortness of breath [1]. In 2000, the prevalence of asthma and wheezing among Swedish children aged 11-12 years were estimated to be 7.7% and 9.4%, respectively [2]. In 2011, the prevalence of physician-diagnosed asthma and exercise-associated dyspnoea among Swedish children aged 12-13 years were reported to be 14.6% and 14.3%, respectively [3].

Adult cross-country skiers are especially prone to having asthma and exercise-induced asthma, and this is assumed to be caused by repeated and intense breathing of dry and cold air [4]. In a recent cross-sectional postal questionnaire study, the asthma prevalence was estimated to be 29–35% in Swedish young adults aged 15–19 years and adult cross-country skiers [5]. The onset of “occupational” asthma in athletes has previously been reported to occur mostly during adulthood [6]. However, according to our recent data, the age of asthma onset among cross-country skiers is predominantly during adolescence [5].

The present study aimed to determine the prevalence of asthma and respiratory symptoms among competitive cross-country skiers in early adolescence in comparison to a reference group. Our hypothesis is that children in early adolescence who compete in cross-country skiing have a higher prevalence of asthma and respiratory symptoms than a population-based reference group of similar ages.

## 2. Materials and Methods

### 2.1. Ethical approval

The study was approved by the Ethical Review Board in Umeå, Sweden.

### 2.2. Study Design

This study is a postal questionnaire survey addressing adolescent skiers from all regions participating in the Swedish National Cross-country Championship together with a random population-based reference population of adolescents with similar ages. Study subjects received a postal questionnaire based on the European Community Respiratory Health Survey II (ECHRSII) [7] and the International Study of Asthma and Allergies in Childhood (ISAAC) [8]. Subjects with physician-diagnosed asthma were asked to complete the Asthma Control Test™ (ACT). The ACT surveys activity limitations, shortness of breath, awakenings with asthma, use of rescue medication, and general asthma control [9].

### 2.3. Study Population

All skiers between the ages of 12–15 years from all geographic regions of Sweden (*n* = 358; 49% female) were invited. Two out of the 20 invited regions did not respond and were therefore excluded.

The reference group consisted of a random population-based sample of adolescents aged 13-14 years (*n* = 1000), matched to the group of skiers by geographical region and sex. Subject information, including addresses, was obtained from the Swedish Population Register.

### 2.4. Questionnaires

The group of skiers received the questionnaire through the team coaches at the Swedish National Championship in February 2016, and the reference group received the postal questionnaire in January and February 2015. Signed consent had to be given by both the guardian and adolescent. One reminder was sent to nonresponders.

The key study variables were as follows: (1) self-reported asthma (physician-diagnosed asthma and use of asthma medication the last 12 months), (2) current wheezing, (3) wheezing without a common cold, (4) exercise-induced wheezing (during the previous 12 months), (5) smoking in family (mother, father, or other person in the family who smokes), (6) use of short-acting/long-acting B2-agonists (SABA/LABA), inhaled corticosteroids (ICS), ICS + LABA, or other medication (“have you used short-acting/long-acting B2-agonists (SABA/LABA), inhaled corticosteroids (ICS), ICS + LABA or other medications (e.g., leukotriene receptor antagonist and cromoglicate) in the last 12 months?”), (7) the presence of allergic rhinitis (report of hay fever or other allergies with rhinitis or conjunctivitis), and (8) exercise (“on average, how many hours per week have you exercised so much that you were out of breath or sweated in the last 12 months?”). The ACT score was a continuous variable ranging from 5 to 25 points, and the cutoff value for controlled asthma was >19 points [9].

### 2.5. Statistical Analysis

The prevalence of asthma in the reference group was assumed to be 8%, and we cautiously expected a response rate of 40%. To be able to show a difference in prevalence between the groups of 7% (alpha 0.05 and beta 0.20), 183 cross-country skiers were needed. Medians and interquartile ranges (IQR) were used for skewed data. Group comparisons were made with a chi-squared test for categorical variables; the Mann–Whitney *U* test and *t*-test were used for continuous variables without and with normal distributions, respectively. Logistic regression analysis was performed to test for an independent association between the prevalence of self-reported asthma and being a skier, after adjustment for exercise level (“low” = those training less than 7 hours/week versus “high” = training 7 hours/week or more). The variable exercise was skewed and therefore dichotomized based on whether or not the subjects reached the international and national guidelines [10] of at least 60 minutes of pulse-raising activity each. A *p*-value <0.05 was considered statistically significant.

## 3. Results

The response rates among skiers and in the reference group were 27% (*n* = 87) and 29% (*n* = 292), respectively. The basic characteristics of the study population are presented in Table 1. A higher proportion of skiers reported asthma and wheezing when compared with the reference group (23% vs. 12%, *p*=0.014, and 25% vs. 14%, *p*=0.017). Skiers were slightly younger and had a higher weekly duration of physical activity than the reference group. Among the skiers, we observed no difference in weekly duration of physical activity among those with asthma compared to those without asthma. In the reference group, boys had a higher duration of weekly physical activity than the girls (*p*=0.016), and subjects with asthma had a higher weekly duration of physical activity compared to those without asthma (median (IQR), 5 (4–8) vs. 4 (3–6) hours/week, *p*=0.014). In the logistic regression model, self-reported asthma was independently associated with being a skier, odds ratio (95% confidence interval) 2.86 (1.30–6.15), but not with high exercise level, 2.10 (0.98–4.34).

A description of and comparison between skiers and references with self-reported asthma are presented in Table 2. Current wheezing, allergic rhinitis, and a family history of asthma were equally common in both groups. Healthcare contacts due to respiratory problems were significantly more common among skiers (50% vs. 11%, *p*=0.001) than in the reference group. Among the skiers with asthma, 25% reported the use of ICS + LABA during the last 12 months. No difference was observed in the use of SABA/LABA, ICS, or ICS + LABA between the two groups, though they were small, nor in the level of asthma control, according to the ACT. The median (IQR) age of asthma onset did not differ between skiers (7 (2–10) years) and the reference group (6 (3–11) years) (*p*=0.891). Among the reference subjects with self-reported asthma, a larger proportion of boys than girls reported current wheezing and had a higher median (IQR) duration of weekly physical activity (83% vs. 46%, *p*=0.023 and 7 (5–10) vs. 4 (3–6) hours/week, *p*=0.016, respectively), although the number of subjects was small. No other comparisons between sexes yielded significant results; however, stratifying by sex contributed to an impaired statistical power.

Among skiers (*n* = 64) and reference subjects (*n* = 248) without self-reported asthma, a low proportion (8% and 6%, respectively) reported wheezing during the last 12 months. In both cross-country skiers and reference subjects, those with current wheezing were predominantly those who had self-reported asthma and a family history of asthma. Subjects who reported current wheezing in the reference group exercised more hours per week than those without wheezing (*p*=0.028) while no such difference was found among cross-country skiers (Table 3).

## 4. Discussion

In this cross-sectional postal survey among Swedish cross-country skiers in early adolescence, the prevalence of self-reported asthma and wheezing during the last 12 months was significantly higher than in a population-based reference group. The prevalence was close to twice as high among cross-country skiers (23% and 25% for self-reported asthma and wheezing, respectively), when compared to the reference group (12% and 14%, respectively).

Asthma among cross-country skiers has almost been considered an occupational disease [6]. It is worrying that an increased prevalence of asthma is already detected among adolescent skiers at the age of 13-14 years. This troubling finding is not unique to cross-country skiing. An increased prevalence of asthma has been detected among competitive swimmers at an early age [11]. We agree with the view that the increased prevalence of asthma among adult and adolescent skiers is due to increased physical activity and increased breathing of cold and dry air leading to dehydration of the airways, which is in line with the presumed pathogenesis of asthma among winter endurance athletes [12]. The present study provides support for these beliefs; skiers exercised more (assessed as hours/week) than the reference group, and in the reference group, respondents with asthma exercised more each week than those without asthma. The results also supports climatic factors as predictors of asthma among athletes, as being a skier was an independent predictor for self-reported asthma irrespective of exercise level. Among those with self-reported asthma in the reference group, boys reported more hours of exercise per week than girls. However, this difference should be interpreted cautiously, as it has been shown that 13- to 15-year-old boys with exercise-induced bronchoconstriction may overestimate their exercise levels [13].

Another explanation for the high prevalence of asthma among skiers is an increased awareness of asthma and the symptoms of asthma among athletes, their parents, coaches, and healthcare personnel. Additionally, it is possible that skiers have higher demands on their respiratory ventilation capacity and therefore experience more and tolerate fewer symptoms. This may also contribute to the fact that the skiers with asthma had more frequent contact with the healthcare than the reference group with asthma in our study.

The skiers in the present study largely fit into the characteristics of those with atopic asthma and do not provide support for the sports asthma phenotype hypothesis. The sports asthma phenotype, in contrast to atopic asthma, involves respiratory symptoms, physical activity, and the absence of allergies, has been suggested to be prevalent among winter and water sports athletes [14].

Overall, skiers reported more respiratory symptoms than the reference group, probably because the skiers had a higher prevalence of asthma, exercised more frequently, and perhaps had an increased exposure to subzero temperatures. Respiratory symptoms, in general, are very common during physical activity in cold temperatures [15].

It is worrying that being an early adolescent skier, even at exercise levels that do not reach the international and national guidelines of at least 60 minutes of pulse-raising activity each day [10], is independently associated with an increased prevalence of self-reported asthma. Still, this does not contradict the current hypothesis that “skiers asthma” is due to repeated and prolonged hyperventilation of cold and dry air, leading to airway injury. In fact, also short-term intense physical exercise in subzero temperatures has been shown to induce acute transient airway obstruction in healthy adult subjects [16].

It is a strength that our survey was based on well-validated questionnaires, ECRHS II and ISAAC [7, 8]. The main limitation of the study was the low response rate, a possible selection bias, and thus impaired validity of the results. One might indeed assume that subjects with asthma or respiratory symptoms would be more inclined to participate in the study, leading to an overestimation of asthma prevalence. Also, according to the official database at the Swedish National Board of Health and Welfare [17] around 60 patients/1000 inhabitants (i.e., 6% of the population) aged 10–14 were prescribed inhaled asthma medication or montelukast during 2015–2016. This may indicate that our results overestimate the prevalence of self-reported asthma. On the other hand, the reference group had similar prevalences of asthma and respiratory symptoms and a similar amount of physical activity as those reported in a previous Swedish epidemiological study in the same age group [3], supporting the assumption that the reference group was representative. Furthermore, a Swedish questionnaire survey with a 62% response rate found no difference in respiratory symptoms and asthma when comparing responders and nonresponders [18]. In a quite recent large population-based study in Norway, the response rate was lower, 33%, comparable to that in our study. Still the prevalences of physician-diagnosed asthma and several respiratory symptoms were similar among responders and nonresponders [19]. A nonresponder analysis was not conducted in our study due to the lack of ethical permission, and we cannot exclude the presence of different selection biases in the cross-country skiers and the reference population. However, the recently performed nonresponder analyses in the Swedish [18] and the Norwegian [19] studies indicate that our results are fairly valid. To further complicate the discussion, the estimation of asthma prevalence through ISAAC questionnaires answered by the children's parents may not always correlate with the actual prevalence, as the parents may not be aware of or observe the children's respiratory symptoms [20], leading to an underestimation of asthma prevalence. The definitions of asthma used in epidemiological studies vary, leading to variable estimates of asthma prevalence [21]. It is a limitation that the asthma diagnosis was self-reported and not validated by medical records, spirometry, or bronchial provocation tests. We cannot exclude misdiagnosis; for instance, exercise-induced laryngeal obstruction may mimic asthma [22]. Taken together, we acknowledge that the study might have overestimated the prevalence of asthma among both skiers and reference subjects. However, we do not find any evidence to conclude that the selection bias differs between groups, and thus the group differences are expected to be fairly valid.

To conclude, our hypothesis was confirmed by this Swedish postal questionnaire survey; the prevalence of asthma and respiratory symptoms among competitive cross-country skiers in early adolescence was higher than in a population-based reference group of similar ages. Even though the low response rate in the study is a limitation, the results are considered fairly representative and should be taken into account. The results indicate that preventive measures, such as more restrictions on exercise when the temperature is below a certain level, may be warranted among cross-country skiers in early adolescence. Furthermore, whether wearing breathing masks prevent or attenuate cold air-induced airway morbidity should be examined.

## Figures and Tables

**Table 1:** Demographic and clinical characteristics of Swedish cross-country skiers in early adolescence and a population-based reference group.

	Cross-country skiers			References			*p*-value^*∗*^
	All *n* = 87	Boys *n* = 41	Girls *n* = 46	All *n* = 292	Boys *n* = 159	Girls *n* = 133	
Age, mean (SD)	12.83 (0.69)	12.73 (0.74)	12.91 (0.63)	13.64 (0.64)	13.68 (0.68)	13.59 (0.59)	**<0.001**
Self-reported asthma	20 (23)	9 (22)	11 (24)	36 (12)	23 (14)	13 (10)	**0.014**
Current wheezing	22 (25)	13 (32)	9 (20)	42 (14)	28 (18)	14 (11)	**0.017**
Wheezing without a common cold	17 (20)	10 (24)	7 (15)	27 (9)	19 (12)	8 (6)	**0.009**
Exercise-induced wheezing	18 (21)	10 (24)	8 (17)	33 (11)	22 (14)	11 (8)	**0.024**
Smoking in family	0 (0)	0 (0)	0 (0)	42 (14)	20 (13)	22 (17)	**<0.001**
BMI, median (IQR)	19 (18–20)	18 (18-19)	19 (18–20)	20 (18–22)	20 (18–21)	20 (18–22)	**<0.001**
Exercise (hours/week), median (IQR)	6 (5–8)	6 (5–8)	6 (5–7)	4 (3–7)	5 (3–7)	4 (2–6)	**<0.001**

Data are presented as *n* (%), unless otherwise stated. Significant *p*-values in bold. SD; standard deviation. IQR; interquartile range. ^*∗*^Comparison between cross-country skiers (all) and reference group members (all).

**Table 2:** Comparison between subjects with self-reported asthma among Swedish cross-country skiers in early adolescence and in a population-based reference group.

	Cross-country skiers			References			*p*-value^*∗*^
	All *n* = 20	Boys *n* = 9	Girls *n* = 11	All *n* = 36	Boys *n* = 23	Girls *n* = 13	
Current wheezing	16 (80)	9 (100)	7 (64)	25 (69)	19 (83)	6 (46)	0.393
Wheezing without a common cold	15 (75)	9 (100)	6 (55)	16 (44)	13 (57)	3 (23)	**0.028**
Exercise-induced wheezing	14 (70)	7 (78)	7 (64)	20 (56)	14 (61)	6 (46)	0.289
Allergic rhinitis	10 (50)	7 (78)	3 (27)	21 (60)	13 (59)	8 (62)	0.472
Parent or sibling with asthma	12 (63)	6 (67)	6 (60)	19 (54)	11 (50)	8 (62)	0.529
Smoking in family	0 (0)	0 (0)	0 (0)	5 (14)	2 (9)	3 (23)	0.076
SABA/LABA^1^	19 (95)	8 (89)	11 (100)	36 (100)	23 (100)	13 (100)	0.176
ICS^2^	13 (65)	6 (67)	7 (64)	21 (58)	14 (61)	7 (54)	0.625
ICS + LABA^3^	5 (25)	1 (11)	4 (36)	7 (19)	6 (26)	1 (8)	0.627
Other medication^4^	7 (35)	2 (22)	5 (45)	5 (14)	4 (17)	1 (8)	0.065
ACT score, median (IQR)	21 (19–23)	20 (18–21)	23 (21–24)	22 (19–24)	22 (19–24)	20 (20–23)	0.535
BMI, median (IQR)	18 (17–19)	18 (18-19)	19 (18–21)	20 (19–21)	20 (20-21)	19 (18–21)	**0.009**
Exercise (hours/week), median (IQR)	6 (5–8)	6 (6-7)	5 (5–7)	5 (4–8)	7 (5–10)	4 (3–6)	0.678

Data are presented as *n* (%), unless otherwise stated. Significant *p*-values in bold. IQR; interquartile range. ^1^Short-acting B2-agonist/long-acting B2-agonist. ^2^Inhaled corticosteroids. ^3^Combination of ICS + LABA. ^4^For example, leukotriene receptor antagonist and cromoglicate. ^*∗*^Comparison between individuals with self-reported asthma among all cross-country skiers and all in the reference group.

**Table 3:** Comparison between subjects with and without current wheezing among Swedish cross-country skiers in early adolescence and in a population-based reference group.

	Cross-country skiers			References		
	Current wheezing *n* = 22	No current wheezing *n* = 65	*p*-value^*∗*^	Current wheezing *n* = 42	No current wheezing *n* = 250	*p*-value^*∗*^
BMI, median (IQR)	18 (18-19)	19 (18–20)	0.668	20 (20-21)	20 (18–22)	0.163
Exercise (hours/week), median (IQR)	6 (5–7)	6 (5–8)	0.850	5 (3–8)	4 (2–6)	**0.028**
Self-reported asthma	16 (73)	4 (6)	**<0.001**	25 (60)	11 (4)	**<0.001**
Parent or sibling with asthma	13 (59)	14 (22)	**0.001**	20 (49)	75 (31)	**0.022**
Smoking in family	0 (0)	0 (0)	NA	6 (14)	36 (14)	0.314

Data are presented as *n* (%), unless otherwise stated. Significant *p*-values in bold. SD; standard deviation. IQR; interquartile range. ^*∗*^Comparison between individuals with current wheezing and no current wheezing within the groups cross-country skiers and references, respectively.

## Data Availability

The data used to support the findings of this study are available from the corresponding author upon request.
